# Coinfection of Dermal Fibroblasts by Human Cytomegalovirus and Human Herpesvirus 6 Can Boost the Expression of Fibrosis-Associated MicroRNAs

**DOI:** 10.3390/microorganisms11020412

**Published:** 2023-02-06

**Authors:** Irene Soffritti, Maria D’Accolti, Clara Maccari, Francesca Bini, Eleonora Mazziga, Maria-Cristina Arcangeletti, Elisabetta Caselli

**Affiliations:** 1 Section of Microbiology, Department of Chemical, Pharmaceutical and Agricultural Sciences and LTTA, University of Ferrara, 44121 Ferrara, Italy; 2 Laboratory of Microbiology and Virology, Department of Medicine and Surgery, University of Parma, 43126 Parma, Italy

**Keywords:** HCMV, HHV-6, coinfection, systemic sclerosis, fibrosis, microRNAs

## Abstract

Tissue fibrosis can affect every type of tissue or organ, often leading to organ malfunction; however, the mechanisms involved in this process are not yet clarified. A role has been hypothesized for Human Cytomegalovirus (HCMV) and Human Herpesvirus 6 (HHV-6) infections as triggers of systemic sclerosis (SSc), a severe autoimmune disease causing progressive tissue fibrosis, since both viruses and antiviral immune responses toward them have been detected in patients. Moreover, HCMV or HHV-6A infection was reported to increase the expression of fibrosis-associated transcriptional factors and miRNAs in human dermal fibroblasts. However, it is unlikely that they have separate effects in the infected host, as both viruses are highly prevalent in the human population. Thus, our study aimed to investigate, by quantitative real-time PCR microarray, the impact of HCMV/HHV-6A coinfection on the expression of pro-fibrotic miRNAs in coinfected cells, compared to the effect of single viruses. The results showed a possible synergistic effect of the two viruses on pro-fibrotic miRNA expression, thus suggesting that HCMV and HHV-6 may enhance each other and cooperate at inducing enhanced miRNA-driven fibrosis. These data may also suggest a possible use of virus-induced miRNAs as novel diagnostic or prognostic biomarkers for SSc and its clinical treatment.

## 1. Introduction

Tissue fibrosis is a pathological feature characterized by the massive accumulation of extracellular matrix (ECM), which can occur in nearly every tissue of the body. It occurs in most chronic inflammatory diseases, and when progressive, the process can lead to scarring, organ malfunction, and death, as seen in several end-stage diseases affecting the kidneys, lungs, and heart. Fibrosis can also influence tumor invasion and metastasis, as well as many chronic autoimmune diseases, including scleroderma or systemic sclerosis (SSc) [1]. Although they have a remarkable impact on morbidity and mortality, the mechanisms of the fibrogenesis process are not yet clarified, and specific therapeutic options targeted to fibrosis pathogenesis are extremely scarce, if not absent.

Among fibrosis-related autoimmune diseases, SSc has a prevalence of 7.2–33.9 per 100,000 individuals in Western countries and affects 2–3 times more females than males [2]. The disease is characterized by complex manifestations, but the pathology always includes three main features: the presence of both humoral and cellular immune system alterations, a severe vasculopathy, and an over-production and deposition of ECM by altered fibroblasts, which are the main target cells involved in the disease, together with endothelial cells (ECs), leading to pronounced tissue and multiorgan fibrosis [3,4,5,6].

To date, the etiological agents of SSc are still unclear, but it is widely accepted that the disease occurs as a result of a multistep and multifactorial process, involving genetic predisposition of the host and environmental factors, the latter including both physicochemical agents and infectious agents [6,7]. Among environmental factors, viral infections have been consistently suggested as possible etiological factors, mainly those which persist in the host and can reactivate in susceptible subjects, such as Human Cytomegalovirus (HCMV) and Human Herpesvirus 6 (HHV-6) [8,9,10,11,12,13,14]. Both viruses belong to the *Herpesviridae* family, *Beta-herpesvirinae* subfamily, and are ubiquitous and highly prevalent in the human population. Primary infection occurs in early childhood, and afterwards, they establish a latent infection in the host, reactivating mostly asymptomatically in the healthy adult, whereas symptomatic reactivations happen in immune-dysregulated hosts, where they have been associated with several autoimmune diseases, including those involving the connective tissue [11,12,15,16,17,18].

An important body of evidence supports the role of HCMV and HHV-6 in SSc etiopathogenesis: both viruses have been found reactivated in the skin of SSc subjects (the HHV-6A species, in particular, has the more marked tissue tropism) [10,19], who also were characterized by a strong activation of antiviral humoral immunity, showing significantly higher amounts of antibodies directed toward viral antigens, such as UL94 (HCMV) and U94 (HHV-6), compared to healthy controls [10,20,21,22,23]. In addition, a molecular mimicry has been observed with anti-UL94 autoantibodies recognizing fibroblasts and EC membrane receptors, resulting in fibroblasts’ activation and the stimulation of EC apoptosis [20,24]. Regarding the cellular immunity, the presence of specific HCMV antigen-driven CD8+ T cells has been observed in SSc patients [9]; an impaired NK response against HHV-6A/B was observed in a subset of SSc patients expressing KIR2DL2 receptor [10].

Both viruses can establish a productive infection in fibroblasts and endothelial cells [8,13,14,25,26,27,28]. The in vitro infection of human primary dermal fibroblasts with HCMV and HHV6 has been shown to have a strong impact on the expression of pro-fibrotic factors [8]. Interestingly, we recently observed that the simultaneous presence of HCMV and HHV-6 determines a more prominent and sustained expression of transcriptional factors associated with fibrosis and apoptosis pathways, compared to that observed in singly infected cells, highlighting for the first time the potential cooperation of these beta-herpesviruses in sclerodermic disease [14].

Indeed, fibrosis can affect every type of tissue or organ, possible leading to organ malfunction and failure; however, the factors modulating and influencing this process are still unclarified. In addition, the hypothesized role of infectious agents and other possible environmental triggers (such as mechanical injury, radiation, and toxic compounds) is not fully elucidated, and there is a lack of validated molecules and markers potentially useful in the diagnosis, prognosis, and therapeutic approach for this group of diseases.

In order to understand the molecular pathways involved in SSc pathogenesis, recent attention has been given to the role of epigenetic factors, and in particular to microRNAs (miRNAs), short RNA sequences 20–23 nucleotides in length that are recognized to have a fundamental importance in the regulation of gene expression at the post-transcriptional level [29,30]. Interestingly, several miRNAs associated with vasculopathy and fibro-proliferative alteration have also been found to be dysregulated in SSc patients compared to controls [31]. In parallel, β-herpesvirus infection has been reported to alter miRNA expression in different tissues and cellular types [32,33], and we recently found that the individual infection with HCMV or HHV-6A could induce a remarkable modulation of miRNAs expressed in human primary dermal fibroblast cells [13]. Based on these considerations, here we aimed to investigate the impact of coinfection by HCMV and HHV-6 on the modulation of fibrosis-associated miRNAs in order to evidence any enhanced effect potentially involved in SSc onset and/or progression. For this purpose, primary human dermal fibroblasts were infected in vitro with individual HCMV or HHV-6A inocula or coinfected simultaneously with HCMV and HHV-6A, and miRNAs levels were evaluated in control uninfected, singly infected, and dually infected cells.

## 2. Materials and Methods

### 2.1. Cell Cultures and Viruses

Primary human dermal fibroblasts derived from the adult skin of a single donor (NHDF-Ad, CC-2511; Lonza, Basel, Switzerland) were cultured in complete fibroblast cell medium (Fibroblast Cell Basal Medium, FCBM), supplemented with 2% fetal bovine serum (FBS), 0.1% r-human fibroblast growth factor-B, 0.1% insulin, 0.1% gentamicin sulphate/amphotericin-B (Clonetics™ FGM™-2 Bullet Kit™; Lonza, Basel, Switzerland), as previously described [13,14,34]. Following the manufacturer’s instructions, fibroblasts were sub-cultivated at around 80% confluence, using a “ReagentPack Subculture Reagent Kit” (Lonza, Basel, Switzerland).

The TB40E HCMV strain (a kind gift from Prof. Thomas Mertens, Ulm University, Ulm, Germany) was propagated in MRC5 fibroblast cells (ECACC 05072101, Merck Life Science, Milan, Italy). Briefly, for HCMV titration, ten-fold serial dilutions of HCMV suspensions were used to infect MRC5 fibroblast monolayers grown in 60 mm Petri dishes, performing the titration in triplicate for each dilution. After adsorption (37 °C for 2 h), virus inocula were removed and replaced with fresh Earle’s modified Minimum Essential Medium with 1% l-glutamine, 1% non-essential amino acids, 10% fetal calf serum, supplemented with 0.6% agarose (Merck KGaA, Darmstadt, Germany). Plates were then incubated at 37 °C for 7 days, then cell monolayers were stained with the vital dye neutral red for 2 h, then the medium was discarded and cells were fixed with 10% formalin for 10 min at room temperature. Plaques were counted and titers expressed as mean PFU values/mL. HCMV virus stock contained 10^9^ PFU/mL.

The U1102 strain of HHV-6A was grown in human J-Jhan T cells. The 6A species of HHV-6 was utilized based on our previous findings showing its presence in the skin of SSc patients, supporting its higher tissue tropism compared to the HHV-6B species [10]. Virus titers were assessed as previously reported [13,35]. The HHV-6A U1102 strain was directly titrated by real-time quantitative PCR (qPCR) targeted to the viral U94 gene after propagation in human lymphoid J-Jhan T cells, as previously described. Briefly, total DNA was extracted from J-Jhan cells at complete cytopathic effect and used to quantify the HHV-6A virus genome number as previously described [13]. HHV-6A virus stock contained 10^10^ genome equivalents/mL. The same viral stocks of HCMV and HHV-6A were used for all infection experiments.

### 2.2. Virus Infection

Cell infection experiments were carried out in primary human dermal fibroblasts by using HCMV (T40E) and HHV-6A (U1102) strains. Infections and coinfections were performed in 90% confluent primary human dermal fibroblasts, using a multiplicity of infection (M.O.I.) corresponding to 0.1 PFU/cell for HCMV and 1.0 genome equivalent/cell for HHV-6A, as previously described [8,13]. Virus adsorption was performed for 2 h at 37 °C, then virus inocula were removed and replaced with complete fibroblast cell medium. Cells were then incubated at 37 °C and collected at 0, 1, 2, 4, and 7 days post-infection (d.p.i.). Briefly, cells were centrifuged at 1000× *g* for 5 min at 4 °C, then cell pellets were washed in PBS to remove any presence of extracellular miRNAs and immediately frozen in liquid nitrogen. Pelletized samples were kept at −80 °C until use. Duplicate samples were analyzed for each experiment.

### 2.3. Nucleic Acid Extraction

Total nucleic acids (TNAs) were extracted from aliquots of 10^6^ infected cells by using the AllPrep DNA/RNA/miRNA kit (Qiagen, Hilden, Germany), which allows the simultaneous isolation of total DNA and RNA, including the miRNA fraction (<200 nucleotides). Extracted TNAs were quantified by spectrophotometric analysis reading at 260/280 nm by using a Nanodrop instrument and kept at −80 °C until analysis. The total extracted RNA, including the miRNA fraction, underwent DNase I treatment (Thermo Fisher Scientific, Waltham, MA, USA) in order to eliminate any DNA contamination. The absence of DNA contamination was checked by PCR amplification of human β-actin gene using 10 ng of extracted RNA as a template.

### 2.4. Virus Quantitation in Infected Cells

The virus presence in infected cells was assessed and quantified by specific quantitative real-time PCR (qPCR), using 100 ng of total extracted DNA as a template. Specifically, the HCMV DNA amount was quantified by the qPCR CMV ELITe MGB^®^ Kit (ELITechGroup, Turin, Italy), designed to detect the HCMV DNA exon 4 region of the immediate-early (IE)1 gene, following the manufacturer’s instructions, as already described [8,14]. HHV-6A presence was assessed by a specific qPCR targeting the U94 viral gene, as previously described [17,35]. In addition, the detection and quantification of the house-keeping human RNase P gene was carried out as a control of nucleic acids’ quality and normalization of viral genome copies per cell number. Both qPCR assays were performed in a 7500 Real-time PCR system (ABI PRISM, Applied BioSystems, Milan, Italy), and results were expressed as number of genome copies per µg of DNA.

### 2.5. miRNA Analysis

The analysis of the expression of miRNA in control uninfected, single-infected, and double-infected fibroblasts was carried out by qPCR microarray. In short, 10 ng aliquots of RNA were retrotranscribed by the miRCURY LNA miRNA RT Kit (Qiagen, Hilden, Germany), according to the manufacturer’s instructions. Then, the cDNA products were analyzed by the LNA miRNA Focus PCR Panel (Qiagen, Hilden, Germany), which allows simultaneous identification and quantification of 84 human microRNAs associated with cell fibrosis. Each panel included six controls of the process (RNA spike-in assays controls, interplate calibrators) and four miRNA reference genes (SNORD44, SNORD38B, SNORD49A, U6 snRNA) to allow data normalization. Each microarray was run on a Quant Studio 5 real-time PCR system (Thermo Fisher Scientific, Milan, Italy), and miRNA data were analyzed with the free Qiagen software (Qiagen Gene Globe, https://geneglobe.qiagen.com/ca/analyze (accessed on 6 January 2023)). Results were expressed as fold-change expression value of infected versus uninfected control cells at each time post-infection, after normalizing for the house-keeping endogenous controls. The analysis threshold was set at 2-fold up- or down-modulation compared to control uninfected cells. Duplicate samples from two independent experiments were analyzed.

Ten miRNAs, among those most up- or down-regulated by coinfection, as judged by microarray analysis, were further analyzed by individual miRCURY LNA miRNA PCR Assays, able to quantify specific miRNAs with high sensitivity using LNA-optimized, SYBR^®^ Green-based miRNA PCR (Qiagen, Hilden Germany). The constitutively expressed cellular miR-RTC and miR-SNORD11 were also included in the analysis as controls.

### 2.6. Statistical Analyses

Paired *t*-test was used to analyze the significance of differential miRNA levels between infected and control cells. Bonferroni correction for multiple comparisons was applied. A *p* value ≤ 0.05 was considered as statistically significant.

## 3. Results

### 3.1. HCMV and HHV-6A Coinfection in Primary Human Dermal Fibroblasts

**Figure 1:**
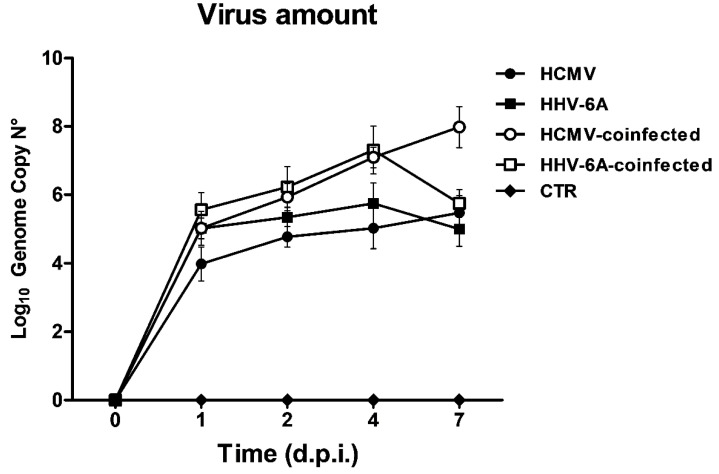
HCMV and HHV-6A replication in single- and double-infected primary human dermal fibroblasts. Results are expressed as Log_10_ of mean values ± S.D. of genome copy number per µg of DNA and are representative of sample duplicates in two independent experiments. HCMV, HCMV single infection; HHV-6A, HHV-6A single infection; HCMV-coinfected, HCMV and HHV-6A coinfected fibroblasts targeting HCMV IE-1 gene; HHV-6A-coinfected, HCMV and HHV-6A coinfected fibroblasts targeting HHV-6A U94 gene; CTR, control uninfected cells.

First, the effect of simultaneous coinfection of HCMV and HHV-6A was analyzed with respect to the ability of viruses to replicate together in human primary dermal fibroblasts. For this purpose, cells were seeded at optimal density in 25 cm^2^ flasks three days before infection, and then they were infected with HCMV (TB40E strain) and HHV-6A (U1102 strain) at a M.O.I. of 0.1 PFU per cell and 1 genome equivalent per cell, respectively. Cell samples from single-infected, double-inflected, and control uninfected fibroblasts were collected at 0, 1, 2, 4, and 7 d.p.i. At each timepoint, total DNA was extracted from collected cell samples and analyzed by specific qPCR targeting HCMV IE-1 and HHV-6 U94 genes, respectively, in order to quantify the amount of the intracellular virus genomes. The results, summarized in Figure 1, confirmed that the primary human dermal fibroblasts used in the experiments were permissive for both HCMV and HHV-6A productive replication, as also previously reported [14], based on the clear increase in viral genome copies inside infected cells over time.

Notably, the genome copy number appeared increased by about 1 Log in coinfected cells with respect to the amount of virus detected in single-infected cells, suggesting that the simultaneous presence of both viruses could enhance their respective replication. This was paralleled by an earlier cytopathic effect (CPE), confirming what was previously reported [14]. In fact, in individually infected fibroblasts, HCMV replication produced a lytic infection with an evident CPE appreciable starting from 4 d.p.i. until the end of the experiment. On the other hand, as also previously reported, HHV-6A did not induce any evident CPE, and likely established a latent infection at 7 d.p.i, as judged by the lack of further increase in virus DNA at this timepoint. Despite the lack of CPE induction by HHV-6A, the simultaneous presence of HCMV induced an earlier HCMV-associated CPE onset, with fibroblasts showing clear morphological alterations as early as 2 d.p.i. (not shown).

### 3.2. Effect of HCMV and HHV-6A Coinfection on the Expression of Fibrosis-Associated miRNAs

**Figure 2:**
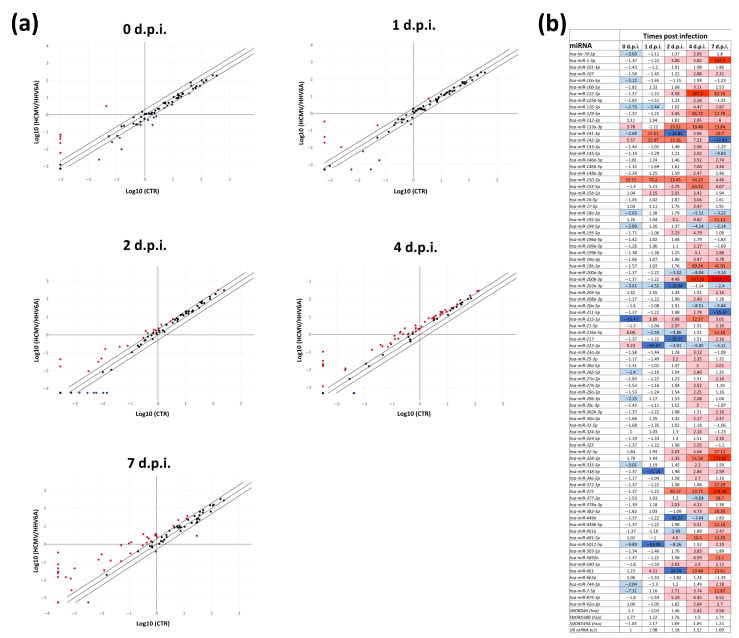
Expression of fibrosis-associated miRNAs in response to HCMV/HHV-6A coinfection of human dermal fibroblasts. Cell samples were collected at the indicated days post-infection (d.p.i.) and analyzed by qPCR microarray. (**a**) Scatterplot representation (threshold put at 2-fold change in coinfected vs. uninfected control cells). Red and blue dots represent up-regulated and down-regulated factors, respectively. Results are expressed as mean values of duplicate samples in two independent experiments. (**b**) Detailed values of down- and up-regulated factors: dark blue, down-regulation >10-fold; light blue, down-regulation between 9.9- and 2-fold; light red, up-regulation between 2- and 9.9-fold; medium red, up-regulation between 10- and 99.9-fold; dark red, up-regulation >100-fold.

The modulation of microRNA expression in control uninfected, individually infected, and coinfected fibroblast cells was analyzed by a specific qPCR microarray able to identify and quantify simultaneously a panel of 84 fibrosis-associated miRNAs. The results (Figure 2) showed a rapid and sustained cell response following HCMV/HHV-6A coinfection, with significant alteration induced in most examined miRNAs at early and late times post-infection. In fact, starting from the moment of virus penetration in the cell at the end of the adsorption time (0 d.p.i.) until the end of the experiment, several miRNAs were found differentially expressed by at least 2-fold compared to uninfected controls at all tested timepoints (0, 1, 2, 4, and 7 d.p.i.).

Specifically, at 0 d.p.i., right after virus adsorption, 19 miRNAs were altered with respect to uninfected control cells. Of these, five miRNAs were up-regulated, including miR-133a (3.78-fold), miR-142 (5.57-fold), miR-150 (52.51-fold), miR-203a (39.38-fold), miR-216a (6.06-fold), and miR-223 (9.23-fold). Fourteen miRNAs were instead down-regulated, some of them slightly above the 2-fold threshold, including miR-let-7d (−2.03-fold), miR-10a (−3.12-fold), miR-126 (−2.73-fold), miR-141 (−2.69-fold), miR-18a (−2.63-fold), miR-194 (−2.89m-fold), miR-203a (−3.61-fold), miR-215 (−15.47-fold), miR-26b (−2.4-fold), miR-29b (−2.15-fold), miR-335 (−3.01-fold), miR-5011 (−9.89-fold), miR-7 (−7.31-fold), and miR-744 (−2.04-fold).

At 1 d.p.i., twelve miRNAs were differentially expressed compared to uninfected controls. They included six miRNAs that were over-expressed at various amounts, comprising miR-141 (15.51-fold), miR-142 (22.97-fold), miR150 (75.2-fold), miR-15b (2.15-fold), miR-203a (66.75-fold), miR-215 (3.89-fold), and miR-661 (4.21-fold). In addition, six miRNAs were instead less expressed compared to controls, including miR-126 (−3.44-fold), miR-203 (−4.52-fold), miR-216a (−2.55-fold), miR-223 (−55.67-fold), miR-338 (−11.24-fold), and miR-5011 (−33.05-fold).

After 48 h of coinfection (at 2 d.p.i.), the number of miRNAs whose expression was altered increased consistently, showing 32 total miRNAs affected by virus coinfection. Among them, 22 miRNAs were up-regulated and 10 were down-regulated compared to controls (Figure 2b). The most over-expressed miRNAs (over 10-fold compared to controls) were miR-133 (15.52-fold), miR-142 (13.16-fold), miR-150 (23.45-fold), and miR-375 (59.17-fold). Among the total down-regulated miRNAs, those most affected (expressed at least −10-fold compared to controls) included miR-141 (−24.82-fold), miR-203a (−18.04-fold), miR-217 (−10.11 fold), miR-449a (−35.23 fold), and miR-661 (−24.29 fold).

At 4 d.p.i., the vast majority of analyzed miRNAs (68 out of the 84 miRNAs included in the qPCR microarray, corresponding to 80.9% of all analyzed miRNAs) exhibited an altered expression in coinfected cells with respect to controls. Interestingly, most of the altered miRNAs showed up-regulated expression, and only seven were down-regulated compared to uninfected cells. Among the many up-regulated miRNAs, the most induced (>10-fold) were represented by miR-122 (187.5-fold), miR-129 (65.72-fold), miR-133 (10.46-fold), miR-150 (54.25-fold), miR-155 (63.32-fold), miR-19b (69.24-fold), miR-200b (337.25-fold), miR-215 (12.37-fold), miR-328 (51.58-fold), miR-375 (23.71-fold), miR-491 (10.5-fold), and miR-661 (12.48-fold). By contrast, no miRNAs were down-regulated more than 10-fold compared to controls, and the degree of modulation ranged from −3.04-fold (miR-449a) to −9.04-fold (miR-377).

Similarly, at the last timepoint analyzed (7 d.p.i.), many of the examined miRNAs (overall, 55/84, 65.5%) appeared over- or under-expressed compared to controls. Again, most of them were up-regulated, whereas only 9 out of the 84 tested miRNAs showed a reduced expression compared to uninfected cells. The most expressed miRNAs included miR-1 (107.3-fold), miR-122 (82.16-fold), miR-129 (17.79-fold), miR-133a (13.84-fold), miR-141 (19.7-fold), miR-192 (11.12-fold), miR-19b (42.93-fold), miR-200b (1321.79-fold), miR-216a (12.16-fold), miR-32 (57.13-fold), miR-328 (173.92-fold), miR-372 (27.29-fold), miR-375 (114.48-fold), miR-377 (28.7-fold), miR-382 (16.35-fold), miR- 449b (12.16-fold), miR-491 (13.35-fold), miR-5692a (11.1-fold), miR-661 (23.61-fold), and miR-7 (11.87-fold). The most down-regulated miRNAs included miR-142 (−12.83-fold) and miR-211 (−35.37-fold), whereas the other miRNAs were expressed between −2.14 and −9.83-fold with respect to controls.

Of note, significant differences were observed by comparing the levels of miRNA expression induced by single and double infection in fibroblast cells (Table S1).

Briefly, three different conditions were observable. For miRNAs that were up-regulated or down-regulated by both individual infections and coinfection, the level of alteration was invariably higher in coinfected cells compared to single-infected cells, supporting the enhancing joint effect of coinfection compared to single infection on miRNA expression. An example of this was miR-1 at 7 d.p.i., which was much more up-regulated by coinfection than by individual viruses. Similarly, the following miRNAs were more induced by coinfection compared to single infections at the indicated times p.i.: miR-122 at 2, 4, and 7 d.p.i.; miR-129 at 4 and 7 d.p.i.; miR-133a at 2, 4, and 7 d.p.i.; miR-150 at all times p.i.; miR-192 at 2, 4, and 7 d.p.i.; miR 19b at 4 and 7 d.p.i.; miR-200b at 7 d.p.i.; miR-216a at 7 d.p.i.; miR-32 at 4 and 7 d.p.i.; miR-328 at 2, 4, and 7 d.p.i.; miR-372 at 4 and 7 d.p.i.; miR-375 at 2, 4, and 7 d.p.i.; miR-449b at 4 and 7 d.p.i.; miR-491 at 2, 4, and 7 d.p.i.; miR-5692a at 4 and 7 d.p.i.; miR-661 at 4 and 7 d.p.i.

In a similar way, miRNAs that were down-regulated by both single and double infection were expressed at a lower level in coinfected cells compared to single-infected cells. These included miR-141 at 2 d.p.i., miR-211 at 7 d.p.i., miR-338 at 1 d.p.i., and miR-5011 at 0, 1, and 2 d.p.i.

Several miRNAs exhibited a biphasic behavior, with different alterations (increase or decrease) at early and late times p.i., and the effect of coinfection could sometimes be opposite to that observed with one or both the infecting viruses individually used. This condition was observed at various timepoints p.i. for the following miRNAs: miR-1, miR-129, miR-142, miR-18a, miR-200a, miR-203a, miR-211, miR-223, miR-449a, miR-5011, and miR-661.

**Figure 3:**
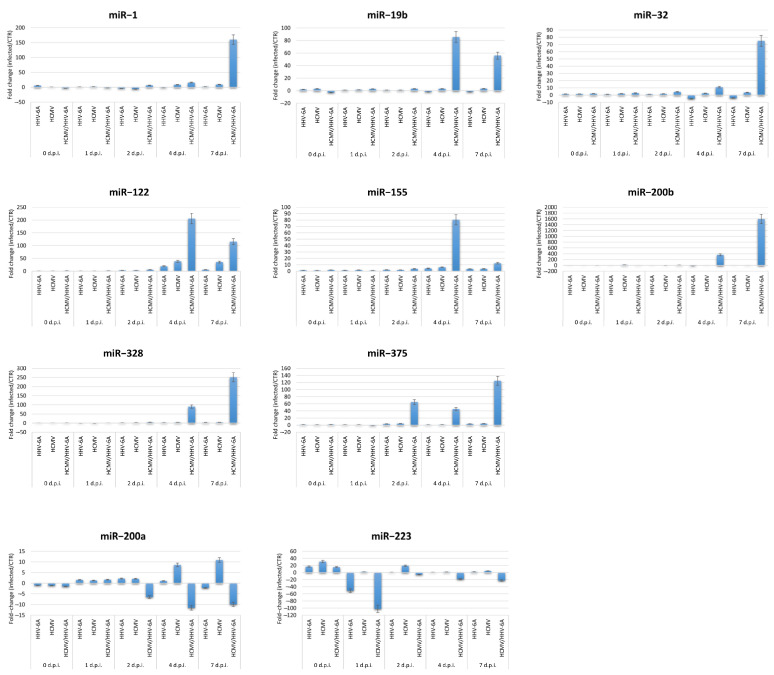
Expression of fibrosis-associated miRNAs most up- or down-regulated by HCMV/HHV-6A coinfection in human dermal fibroblasts. Cell samples were collected at the indicated days post-infection (d.p.i.) and analyzed by individual miRNA assays. Results are expressed as mean values of fold-change ± S.D. of duplicate samples in two independent experiments.

Among the most up- and down-regulated miRNAs in coinfected cells, 10 miRNAs were further analyzed by individual assay to validate and verify the alterations observed by microarray analysis. Specifically, miR-1, miR-122, miR-19b, miR-200b, miR-32, miR-328, miR-375, and miR-155 were tested by individual assay to confirm the up-regulation and miR-200a and miR-223 for down-regulation by coinfection, as observed by microarray analysis. Two control constitutively expressed miRNAs were also included in the analysis as controls (miR-RTC and miR-SNORD11). The results, after normalization for the house-keeping miRNAs, confirmed the data obtained by microarray, indeed showing in most cases superimposable or more significant levels of variations compared to that observed by microarray qPCR (Figure 3).

## 4. Discussion

The beta-herpesviruses HCMV and HHV-6 have been repeatedly hypothesized to have a role in the onset and/or progression of SSc, based on virological and immunological clues. Both viruses are highly prevalent and ubiquitous in the human population, and their reactivation in a susceptible host has been associated with the development of several symptomatic diseases, including the onset of diverse autoimmune diseases, where their concurrent reactivation has been invariably correlated with a worse clinical outcome [36,37,38,39].

Indeed, individual infection by HCMV or HHV-6A in human dermal fibroblasts, which represent the main target cells of SSc disease, has been shown to produce a remarkable impact on the expression of cell factors involved in the progression toward tissue fibrosis [8]. Last, the simultaneous presence of these viruses in coinfected fibroblast cells has been recently reported to induce a higher, pronounced, and sustained expression of cell factors correlated with fibrosis and apoptosis processes compared with single-infected cells [14].

With regard to cell fibrosis, several miRNAs have been reported to be involved in key pathways linked to fibrotic alterations; they have been detected to be significantly altered in tissues and blood of SSc patients, and their expression was similarly found to be modulated in human fibroblasts infected with HCMV or HHV-6 [13,40].

Based on the observation that it is unlikely that these viruses infect or reactivate separately in the host and that currently there are no data on their joint effect on miRNA expression in coinfected cells, our study aimed to investigate the modulation of miRNA expression following coinfection in primary human dermal fibroblasts, comparing it with control uninfected cells and individually infected cells.

The intracellular miRNA profiling of 84 fibrosis-related miRNAs by microarray evidenced a huge enhancing effect on miRNA expression in HCMV/HHV-6A coinfection compared to what was observed in single-infected cells. This was likely correlated to the reciprocal boost of virus replication associated with the simultaneous presence of both viruses, as demonstrated by the 1–2 Log increase in viral genome copy number in coinfected cells compared to what was detected in single-infected cells, despite the equal amount of viral inocula used. This highlights that HCMV and HHV-6 can enhance each other’s replication and supports previous reported data on the observed synergism of beta-herpesviruses in the induction of serious clinical manifestation and worse patient outcome [38,41,42].

Consistently, the co-presence of HCMV and HHV-6A resulted in a strengthened effect on miRNA expression. In fact, a higher level of dysregulation of miRNA expression was detected compared to single-infected cells for several fibrosis-associated miRNAs. Specifically, the pro-fibrotic miR-1, miR-19b; miR-122, miR-129, miR-150, miR-155, miR-192, miR-200b, miR-215, miR-216a, miR-32, miR-328, miR-375, miR-449b, miR-491, miR-661, and miR-7 were all more induced than in single-infected cells, showing values suggesting true synergism between the viruses rather than a mere additional effect. The pro-fibrotic action of the up-regulated miRNAs was well documented in several reports; miR-1 was reported as involved in increased fibrosis of the cartilage in acetabular dysplasia [43] and in liver fibrosis [44]; miR-19b-3p was associated with hypertrophic and fibrosis indexes in acute heart failure [45]; miR-122 has been related with fibrosis-induced cardiovascular remodeling [46] and, in this regard, it is intriguing that both HCMV and HHV-6A have been associated with cardiovascular diseases [25,47,48,49]; miR-150 was correlated with renal fibrosis and miR-150 antagonists can ameliorate the pro-fibrotic pathway in a mouse model [50]; miR-155 is essential in fibrosis and it is consistently up-regulated in fibrotic disorders [51]; miR-192 can promote fibrosis by transforming growth factor beta (TGF-β) activation, which is recognized as a major mediator of fibrosis [52], and its circulating levels are increased in patients with hypertrophic cardiomyopathy and diffuse myocardial fibrosis [53]; miR-200b was found to be up-regulated in fibrotic liver samples compared to non-fibrotic ones [54]; miR-216a accelerates fibrogenesis in cardiac fibroblasts [55]; miR-32 has been reported to mediate the glucose-induced hepatic fibrosis [56]); miR-328 was found to be up-regulated in cardiac fibrosis and shown to directly stimulate TGF-β1 signaling, promoting collagen production in cultured fibroblasts [57], although its pro-fibrotic role it is not so clear, since it has been also reported to prevent renal fibrogenesis [58]; miR-375 was recognized to promote cardiac fibrogenesis by accelerating the ferroptosis of cardiomyocytes through mediating glutathione peroxidase 4 (GPX4) [59]; the miR-449 family was detected to be up-regulated in cystic fibrosis [60] and in bleomycin-induced lung fibrosis [61] and able to activate TGF-β1 in nasopharyngeal carcinoma [62]; miR-491 is induced by TGF-β1 during renal fibrosis [63]; miR-661 has been recently shown to accelerate fibrosis by increasing fibroblast growth factor 2 (FGF2) [64]; miR-7, besides its recognized role as a tumor suppressor in the liver, has been recently reported to promote Fibroblast growth factor receptor 4 (FGFR4) activation and associated liver fibrosis [65].

Consistent with the hypothesized pro-fibrotic action of HCMV/HHV-6A coinfection, it concomitantly decreased the expression of miRNAs with putative or recognized anti-fibrotic effect, such as miR-145, miR-18a, miR-194, miR-200a, miR-223, miR-338, and miR-449a. Other antifibrotic miRNAs were instead variably regulated or indeed induced by HCMV/HHV-6A coinfection. For example, miR-215 was down-regulated by coinfection at earlier times p.i. but up-regulated at later times, and published data suggest an anti-fibrotic action, showing that its dampening correlates with increased fibroblastic production of matrix in ocular pterygium [55]. Similarly, miR-133a, up-regulated by coinfection, despite its induction by TGF-β1, was identified as an anti-fibrotic factor functioning as a feedback negative regulator of TGF-β1 pro-fibrogenic pathways [66]. In addition, miR-141 was reported to reduce cardiac fibrosis and improve cardiac function [67]. The results were also in agreement with previous published results obtained in individually infected cells by a microRNA two-card array set able to identify and quantify simultaneously a total of 754 miRNA by qPCR TaqMan assays [13]. Most of the differentially expressed miRNAs in the present study were in fact also identified in the previous analysis, among those up-regulated or down-regulated by HCMV or HHV-6A in in vitro infected primary human dermal fibroblasts. These included miR-1, miR-19b, miR-122, miR-192, miR-200b, miR-32, miR-449b, and miR-7, which were also among the most up-regulated by individual infections [13]. By contrast, miR-150, miR-155, miR-215, miR-216a, miR-328, miR-375, miR-491, and miR-661 appeared noticeably more induced only by coinfection, highlighting the synergistic effects related to the simultaneous presence of both HCMV and HHV-6A, rather than a mere summation effect. Quantitative differences observed in single-infected cells in the two studies are likely due to the different methodologies used. However, despite the diverse analytical method employed, the results confirmed what was previously reported, underlining the reliability of the microarray results obtained here, as well as the significant differences that emerged between single- and double-infected fibroblasts in terms of the modulation of their microRNome.

Although it is difficult to draw conclusions on the impact of virus coinfection on a possible miRNA-mediated fibrosis, it is interesting to note that we previously demonstrated that HCMV/HHV-6A simultaneous infection increased TNFα and other fibrosis-associated factors and that miR-19b and miR-155, both induced by coinfection, were reported to trigger endothelial IL-6 and TNFα production [68]. In addition, miR-129 was reported to be involved in an IL-6/TNFα signaling axis in neuroinflammation [69]. miR-150 overexpression promotes cell apoptosis, inhibited cell proliferation, and increased secretion of pro-inflammatory cytokines such as IFNγ and TNFα [70]. miR-155 may play an important role in the pathogenesis of fibrosis by pro-inflammatory factors IL-1β and TNFα [71], by which it is also induced [72]. MiR-32 could enhance cell proliferation and epithelial-to-mesenchymal transition via an miR-32/CXCR4 axis [73], and CXCR4 was found to be up-regulated by HCMV/HHV-6A coinfection. On the other hand, miR-200a, found to be down-regulated by virus coinfection, was reported to suppress epithelial-to-mesenchymal transition via BMP7 [74], another factor significantly affected by virus coinfection [14]. Similarly, miR-223 is considered a regulator of inflammation via CCL3 and CXCR4 regulation [75,76], which were instead detected to be overexpressed by us in coinfected cells [14], consistent with miR-223 down-regulation. Other signaling pathways appear less clear and somehow contradictory, such as for example those related to the induction of miR-1 and miR-19b, which have been both reported to decrease the expression of CXCR4 [77,78], rather than enhancing its production. Overall, functional studies focused on the most important factors and miRNAs impacted by HCMV/HHV-6A coinfection would be needed to assess which pathways are responsible for the hypothesized virus-induced pro-fibrotic effect.

## 5. Conclusions

Tissue fibrosis is a complex and multistep process, involving several cellular factors and signaling. Among them, miRNA expression has been recognized as a crucial mechanism underlying the development of fibrosis. Our data show that HCMV/HHV-6A coinfection, which is a highly probable condition in vivo, can boost the expression of miRNAs associated with fibrosis compared to individual viruses [13], strengthening the hypothesis of a significant role of the simultaneous presence of these two beta-herpesviruses in fibrosis-associated diseases, such as SSc. Of note, only some of the induced pro-fibrotic miRNAs have been already recognized to be altered in SSc patients’ tissues or blood (such as miR-133, miR-145, and miR-223), while several others have been associated with other fibrosis-associated diseases, extending the potential putative involvement of HCMV and HHV-6A virus coinfection in more fibrosis-related pathologies. Indeed, the data highlighted a high induction of miRNAs involved in cardiovascular diseases, supporting the hypothesized role of these viruses in the pathogenesis of cardiac and vascular troubles, and suggesting their role as possible triggers of fibrosis for these specific tissues. Of note, miRNA deregulation also was consistent with previously reported data on the expression of cellular factors associated with the fibrosis process. In conclusion, these data show for the first time that coinfection by HCMV and HHV-6A can result in high and sustained dysregulation of miRNA expression in coinfected cells, thus potentially opening the way to considering the use of these virus-induced miRNAs as novel diagnostic or prognostic biomarkers for SSc, as well as for other fibrosis-related diseases, with potential usefulness in the assessment of disease onset and progression to differentiate and foresee the diverse clinical outcomes.

## Data Availability

All the data supporting reported results are included in the present paper and in the Supplementary Materials.

## Supplementary Materials

The following supporting information can be downloaded at: https://www.mdpi.com/article/10.3390/microorganisms11020412/s1, Table S1: miRNA expression in single- and dual-infected cells (*).

## References

[1] Wynn T.A., Ramalingam T.R. (2012). Mechanisms of Fibrosis: Therapeutic Translation for Fibrotic Disease. Nat. Med..

[2] Bergamasco A., Hartmann N., Wallace L., Verpillat P. (2019). Epidemiology of Systemic Sclerosis and Systemic Sclerosis-Associated Interstitial Lung Disease. Clin. Epidemiol..

[3] Denton C.P., Khanna D. (2017). Systemic Sclerosis. Lancet.

[4] Wigley F.M., Boin F. (2017). Clinical Features and Treatment of Scleroderma. Kelley and Firestein’s Textbook of Rheumatology.

[5] Ferri C., Sebastiani M., Lo Monaco A., Iudici M., Giuggioli D., Furini F., Manfredi A., Cuomo G., Spinella A., Colaci M. (2014). Systemic Sclerosis Evolution of Disease Pathomorphosis and Survival. Our Experience on Italian Patients’ Population and Review of the Literature. Autoimmun. Rev..

[6] Ferri C., Arcangeletti M.-C., Caselli E., Zakrzewska K., Maccari C., Calderaro A., D’accolti M., Soffritti I., Arvia R., Sighinolfi G. (2021). Insights into the Knowledge of Complex Diseases: Environmental Infectious/Toxic Agents as Potential Etiopathogenetic Factors of Systemic Sclerosis. J. Autoimmun..

[7] Murdaca G., Contatore M., Gulli R., Mandich P., Puppo F. (2016). Genetic Factors and Systemic Sclerosis. Autoimmun. Rev..

[8] Arcangeletti M.C., D’accolti M., Maccari C., Soffritti I., De Conto F., Chezzi C., Calderaro A., Ferri C., Caselli E. (2020). Impact of Human Cytomegalovirus and Human Herpesvirus 6 Infection on the Expression of Factors Associated with Cell Fibrosis and Apoptosis: Clues for Implication in Systemic Sclerosis Development. Int. J. Mol. Sci..

[9] Arcangeletti M.C., Maccari C., Vescovini R., Volpi R., Giuggioli D., Sighinolfi G., De Conto F., Chezzi C., Calderaro A., Ferri C. (2018). A Paradigmatic Interplay between Human Cytomegalovirus and Host Immune System: Possible Involvement of Viral Antigen-Driven CD8+ T Cell Responses in Systemic Sclerosis. Viruses.

[10] Caselli E., Soffritti I., D’Accolti M., Bortolotti D., Rizzo R., Sighinolfi G., Giuggioli D., Ferri C. (2019). HHV-6A Infection and Systemic Sclerosis: Clues of a Possible Association. Microorganisms.

[11] Broccolo F., Drago F., Paolino S., Cassina G., Gatto F., Fusetti L., Matteoli B., Zaccaria E., Parodi A., Lusso P. (2009). Reactivation of Human Herpesvirus 6 (HHV-6) Infection in Patients with Connective Tissue Diseases. J. Clin. Virol..

[12] Broccolo F., Drago F., Cassina G., Fava A., Fusetti L., Matteoli B., Ceccherini-Nelli L., Sabbadini M.G., Lusso P., Parodi A. (2013). Selective Reactivation of Human Herpesvirus 6 in Patients with Autoimmune Connective Tissue Diseases. J. Med. Virol..

[13] Soffritti I., D’Accolti M., Ravegnini G., Arcangeletti M.C., Maccari C., De Conto F., Calderaro A., Caselli E. (2021). Modulation of Micrornome by Human Cytomegalovirus and Human Herpesvirus 6 Infection in Human Dermal Fibroblasts: Possible Significance in the Induction of Fibrosis in Systemic Sclerosis. Cells.

[14] Soffritti I., D’Accolti M., Maccari C., Bini F., Mazziga E., de Conto F., Calderaro A., Arcangeletti M.C., Caselli E. (2022). Human Cytomegalovirus and Human Herpesvirus 6 Coinfection of Dermal Fibroblasts Enhances the Pro-Inflammatory Pathway Predisposing to Fibrosis: The Possible Impact on Systemic Sclerosis. Microorganisms.

[15] Álvarez-Lafuente R., Fernández-Gutiérrez B., De Miguel S., Jover J.A., Rollin R., Loza E., Clemente D., Lamas J.R. (2005). Potential Relationship between Herpes Viruses and Rheumatoid Arthritis: Analysis with Quantitative Real Time Polymerase Chain Reaction. Ann. Rheum. Dis..

[16] Alvarez-Lafuente R., Martinez A., Garcia-Montojo M., Mas A., De Las Heras V., Dominguez-Mozo M.I., Maria Del Carmen C., López-Cavanillas M., Bartolome M., Gomez De La Concha E. (2010). MHC2TA Rs4774C and HHV-6A Active Replication in Multiple Sclerosis Patients. Eur. J. Neurol..

[17] Caselli E., Zatelli M.C., Rizzo R., Benedetti S., Martorelli D., Trasforini G., Cassai E., degli Uberti E.C., Di Luca D., Dolcetti R. (2012). Virologic and Immunologic Evidence Supporting an Association between HHV-6 and Hashimoto’s Thyroiditis. PLoS Pathog..

[18] Magro C.M., Crowson A.N., Ferri C. (2007). Cytomegalovirus-Associated Cutaneous Vasculopathy and Scleroderma sans Inclusion Body Change. Hum. Pathol..

[19] Ferri C., Cazzato M., Giuggioli D., Sebastiani M., Magro C. (2002). Systemic Sclerosis Following Human Cytomegalovirus Infection. Ann. Rheum. Dis..

[20] Lunardi C., Dolcino M., Peterlana D., Bason C., Navone R., Tamassia N., Beri R., Corrocher R., Puccetti A. (2006). Antibodies against Human Cytomegalovirus in the Pathogenesis of Systemic Sclerosis: A Gene Array Approach. PLoS Med..

[21] Arnson Y., Amital H., Guiducci S., Matucci-Cerinic M., Valentini G., Barzilai O., Maya R., Shoenfeld Y. (2009). The Role of Infections in the Immunopathogensis of Systemic Sclerosis-Evidence from Serological Studies. Ann. N. Y. Acad. Sci..

[22] Marou E., Liaskos C., Efthymiou G., Dardiotis E., Daponte A., Scheper T., Meyer W., Hadjigeorgiou G., Bogdanos D.P., Sakkas L.I. (2017). Increased Immunoreactivity against Human Cytomegalovirus UL83 in Systemic Sclerosis. Clin. Exp. Rheumatol..

[23] Efthymiou G., Dardiotis E., Liaskos C., Marou E., Scheper T., Meyer W., Daponte A., Daoussis D., Hadjigeorgiou G., Bogdanos D.P. (2019). A Comprehensive Analysis of Antigen-Specific Antibody Responses against Human Cytomegalovirus in Patients with Systemic Sclerosis. Clin. Immunol..

[24] Lunardi C., Bason C., Navone R., Millo E., Damonte G., Corrocher R., Puccetti A. (2000). Systemic Sclerosis Immunoglobulin G Autoantibodies Bind the Human Cytomegalovirus Late Protein UL94 and Induce Apoptosis in Human Endothelial Cells. Nat. Med..

[25] Rotola A., Di Luca D., Cassai E., Ricotta D., Giulio A., Turano A., Caruso A., Muneretto C. (2000). Human Herpesvirus 6 Infects and Replicates in Aortic Endothelium. J. Clin. Microbiol..

[26] Caruso A., Rotola A., Comar M., Favilli F., Galvan M., Tosetti M., Campello C., Caselli E., Alessandri G., Grassi M. (2002). HHV-6 Infects Human Aortic and Heart Microvascular Endothelial Cells, Increasing Their Ability to Secrete Proinflammatory Chemokines. J. Med. Virol..

[27] Mostmans Y., Cutolo M., Giddelo C., Decuman S., Melsens K., Declercq H., Vandecasteele E., De Keyser F., Distler O., Gutermuth J. (2017). The Role of Endothelial Cells in the Vasculopathy of Systemic Sclerosis: A Systematic Review. Autoimmun. Rev..

[28] Sinzger C., Grefte A., Plachter B., Gouw A.S.H., Hauw The T., Jahn G. (1995). Fibroblasts, Epithelial Cells, Endothelial Cells and Smooth Muscle Cells Are Major Targets of Human Cytomegalovirus Infection in Lung and Gastrointestinal Tissues. J. Gen. Virol..

[29] Altorok N., Almeshal N., Wang Y., Kahaleh B. (2015). Epigenetics, the Holy Grail in the Pathogenesis of Systemic Sclerosis. Rheumatology.

[30] Szabo I., Muntean L., Crisan T., Rednic V., Sirbe C., Rednic S. (2021). Novel Concepts in Systemic Sclerosis Pathogenesis: Role for MiRNAs. Biomedicines.

[31] Henry T.W., Mendoza F.A., Jimenez S.A. (2019). Role of MicroRNA in the Pathogenesis of Systemic Sclerosis Tissue Fibrosis and Vasculopathy. Autoimmun. Rev..

[32] Rizzo R., Soffritti I., D’Accolti M., Bortolotti D., Di Luca D., Caselli E. (2017). HHV-6A/6B Infection of NK Cells Modulates the Expression of MiRNAs and Transcription Factors Potentially Associated to Impaired NK Activity. Front. Microbiol..

[33] Caselli E., Bortolotti D., Marci R., Rotola A., Gentili V., Soffritti I., D’Accolti M., Lo Monte G.L., Sicolo M., Barao I. (2017). HHV-6A Infection of Endometrial Epithelial Cells Induces Increased Endometrial NK Cell-Mediated Cytotoxicity. Front. Microbiol..

[34] Doridot L., Jeljeli M., Chêne C., Batteux F. (2019). Implication of Oxidative Stress in the Pathogenesis of Systemic Sclerosis via Inflammation, Autoimmunity and Fibrosis. Redox Biol..

[35] Caselli E., Bracci A., Galvan M., Boni M., Rotola A., Bergamini C., Cermelli C., Dal Monte P., Gompels U.A., Cassai E. (2006). Human Herpesvirus 6 (HHV-6) U94/REP Protein Inhibits Betaherpesvirus Replication. Virology.

[36] DesJardin J.A., Gibbons L., Cho E., Supran S.E., Falagas M.E., Werner B.G., Snydman D.R. (1998). Human Herpesvirus 6 Reactivation Is Associated with Cytomegalovirus Infection and Syndromes in Kidney Transplant Recipients at Risk for Primary Cytomegalovirus Infection. J. Infect. Dis..

[37] Van Leer-Buter C.C., Sanders J.S.F., Vroom H.E.J., Riezebos-Brilman A., Niesters H.G.M. (2013). Human Herpesvirus-6 DNAemia Is a Sign of Impending Primary CMV Infection in CMV Sero-Discordant Renal Transplantations. J. Clin. Virol..

[38] Handous I., Achour B., Marzouk M., Rouis S., Hazgui O., Brini I., Khelif A., Hannachi N., Boukadida J. (2020). Co-Infections of Human Herpesviruses (CMV, HHV-6, HHV-7 and EBV) in Non-Transplant Acute Leukemia Patients Undergoing Chemotherapy. Virol. J..

[39] Roa P.L., Hill J.A., Kirby K.A., Leisenring W.M., Huang M.L., Santo T.K., Jerome K.R., Boeckh M., Limaye A.P. (2015). Coreactivation of Human Herpesvirus 6 and Cytomegalovirus Is Associated With Worse Clinical Outcome in Critically Ill Adults. Crit. Care Med..

[40] Von Kietzell K., Pozzuto T., Heilbronn R., Grössl T., Fechner H., Weger S. (2014). Antibody-Mediated Enhancement of Parvovirus B19 Uptake into Endothelial Cells Mediated by a Receptor for Complement Factor C1q. J. Virol..

[41] Mendez J.C., Dockrell D.H., Espy M.J., Smith T.F., Wilson J.A., Harmsen W.S., Ilstrup D., Paya C.V. (2001). Human β-Herpesvirus Interactions in Solid Organ Transplant Recipients. J. Infect. Dis..

[42] Humar A., Malkan G., Moussa G., Greig P., Levy G., Mazzulli T. (2000). Human Herpesvirus—6 Is Associated with Cytomegalovirus Reactivation in Liver Transplant Recipients. J. Infect. Dis..

[43] Ding R., Liu X., Zhang J., Yuan J., Zheng S., Cheng X., Jia J. (2021). Downregulation of MiR-1-3p Expression Inhibits the Hypertrophy and Mineralization of Chondrocytes in DDH. J. Orthop. Surg. Res..

[44] Zhang H., Zhang Z., Gao L., Qiao Z., Yu M., Yu B., Yang T. (2019). MiR-1-3p Suppresses Proliferation of Hepatocellular Carcinoma through Targeting SOX9. Onco. Targets. Ther..

[45] Su Y., Sun Y., Tang Y., Li H., Wang X., Pan X., Liu W., Zhang X., Zhang F., Xu Y. (2021). Circulating Mir-19b-3p as a Novel Prognostic Biomarker for Acute Heart Failure. J. Am. Heart Assoc..

[46] Liu Y., Song J.W., Lin J.Y., Miao R., Zhong J.C. (2020). Roles of MicroRNA-122 in Cardiovascular Fibrosis and Related Diseases. Cardiovasc. Toxicol..

[47] Gómez E., Laurés A., Baltar J.M., Melón S., Díez B., De Oña M. (2005). Cytomegalovirus Replication and “Herpesvirus Burden” as Risk Factor of Cardiovascular Events in the First Year After Renal Transplantation. Transplant. Proc..

[48] Horváth R., Černý J., Benedík J., Hökl J., Jelínková I., Benedík J. (2000). The Possible Role of Human Cytomegalovirus (HCMV) in the Origin of Atherosclerosis. J. Clin. Virol..

[49] Comar M., D’Agaro P., Campello C., Poli A., Breinholt J.P., Towbin J.A., Vatta M. (2009). Human Herpes Virus 6 in Archival Cardiac Tissues from Children with Idiopathic Dilated Cardiomyopathy or Congenital Heart Disease. J. Clin. Pathol..

[50] Hao X., Luan J., Jiao C., Ma C., Feng Z., Zhu L., Zhang Y., Fu J., Lai E., Zhang B. (2022). LNA-Anti-MiR-150 Alleviates Renal Interstitial Fibrosis by Reducing pro-Inflammatory M1/M2 Macrophage Polarization. Front. Immunol..

[51] Eissa M.G., Artlett C.M. (2019). The MicroRNA MiR-155 Is Essential in Fibrosis. Non-Coding RNA.

[52] Chung A.C.K., Huang X.R., Meng X., Lan H.Y. (2010). MiR-192 Mediates TGF-β/Smad3-Driven Renal Fibrosis. J. Am. Soc. Nephrol..

[53] Ren F.J., Yao Y., Cai X.Y., Fang G.Y. (2021). Emerging Role of MiR-192-5p in Human Diseases. Front. Pharmacol..

[54] Ye M., Wang S., Sun P., Qie J. (2021). Integrated MicroRNA Expression Profile Reveals Dysregulated MiR-20a-5p and MiR-200a-3p in Liver Fibrosis. Biomed Res. Int..

[55] Lan W., Chen S., Tong L. (2015). MicroRNA-215 Regulates Fibroblast Function: Insights from a Human Fibrotic Disease. Cell Cycle.

[56] Li Q., Li Z., Lin Y., Che H., Hu Y., Kang X., Zhang Y., Wang L., Zhang Y. (2022). High glucose promotes hepatic fibrosis via miR-32/MTA3-mediated epithelial-to-mesenchymal transition Corrigendum in/10.3892/mmr. 2022.12827. Mol. Med. Rep..

[57] Du W., Liang H., Gao X., Li X., Zhang Y., Pan Z., Li C., Wang Y., Liu Y., Yuan W. (2016). MicroRNA-328, a Potential Anti-Fibrotic Target in Cardiac Interstitial Fibrosis. Cell. Physiol. Biochem..

[58] He W., Zhuang J., Zhao Z.G., Luo H., Zhang J. (2018). MiR-328 Prevents Renal Fibrogenesis by Directly Targeting TGF-Β2. Bratisl. Lek. Listy.

[59] Zhuang Y., Yang D., Shi S., Wang L., Yu M., Meng X., Fan Y., Zhou R., Wang F. (2022). MiR-375-3p Promotes Cardiac Fibrosis by Regulating the Ferroptosis Mediated by GPX4. Comput. Intell. Neurosci..

[60] Pommier A., Varilh J., Bleuse S., Delétang K., Bonini J., Bergougnoux A., Brochiero E., Koenig M., Claustres M., Taulan-Cadars M. (2021). MiRNA Repertoires of Cystic Fibrosis Ex Vivo Models Highlight MiR-181a and MiR-101 That Regulate WISP1 Expression. J. Pathol..

[61] Xie T., Liang J., Guo R., Liu N., Noble P.W., Jiang D. (2011). Comprehensive MicroRNA Analysis in Bleomycin-Induced Pulmonary Fibrosis Identifies Multiple Sites of Molecular Regulation. Physiol. Genomics.

[62] Bissey P.A., Law J.H., Bruce J.P., Shi W., Renoult A., Chua M.L.K., Yip K.W., Liu F.F. (2018). Dysregulation of the MiR-449b Target TGFBI Alters the TGFβ Pathway to Induce Cisplatin Resistance in Nasopharyngeal Carcinoma. Oncogenesis.

[63] Chung A.C.K., Lan H.Y. (2015). MicroRNAs in Renal Fibrosis. Front. Physiol..

[64] Wu F., He H., Chen Y., Zhu D., Jiang T., Wang J. (2022). CircPDE7B/MiR-661 Axis Accelerates the Progression of Human Keloid Fibroblasts by Upregulating Fibroblast Growth Factor 2 (FGF2). Mol. Cell. Biochem..

[65] Tian S., Chen M., Wang B., Han Y., Shang H., Chen J., Chen J. (2020). MiR-7-5p Promotes Hepatic Stellate Cell Activation by Targeting Fibroblast Growth Factor Receptor 4. Gastroenterol. Res. Pract..

[66] Wei P., Xie Y., Abel P.W., Huang Y., Ma Q., Li L., Hao J., Wolff D.W., Wei T., Tu Y. (2019). Transforming Growth Factor (TGF)-Β1-Induced MiR-133a Inhibits Myofibroblast Differentiation and Pulmonary Fibrosis. Cell Death Dis..

[67] Li G., Zhao C., Fang S. (2021). SGLT2 Promotes Cardiac Fibrosis Following Myocardial Infarction and Is Regulated by MiR-141. Exp. Ther. Med..

[68] Song T., Zhou M., Li W., Lv M., Zheng L., Zhao M. (2022). The Anti-Inflammatory Effect of Vasoactive Peptides from Soybean Protein Hydrolysates by Mediating Serum Extracellular Vesicles-Derived MiRNA-19b/CYLD/TRAF6 Axis in the Vascular Microenvironment of SHRs. Food Res. Int..

[69] Singh V., Kushwaha S., Ansari J.A., Gangopadhyay S., Mishra S.K., Dey R.K., Giri A.K., Patnaik S., Ghosh D. (2022). MicroRNA-129-5p-Regulated Microglial Expression of the Surface Receptor CD200R1 Controls Neuroinflammation. J. Biol. Chem..

[70] Xia S., Huang J., Yan L., Han J., Zhang W., Shao H., Shen H., Wang J., Wang J., Tao C. (2022). MiR-150 Promotes Progressive T Cell Differentiation via Inhibiting FOXP1 and RC3H1. Hum. Immunol..

[71] Yang L.-L., Liu J.-Q., Bai X.-Z., Fan L., Han F., Jia W.-B., Su L.-L., Shi J.-H., Tang C.-W., Hu D.-H. (2014). Acute Downregulation of MiR-155 at Wound Sites Leads to a Reduced Fibrosis through Attenuating Inflammatory Response. Biochem. Biophys. Res. Commun..

[72] Pottier N., Maurin T., Chevalier B., Puisségur M.-P., Lebrigand K., Robbe-Sermesant K., Bertero T., Lino Cardenas C.L., Courcot E., Rios G. (2009). Identification of Keratinocyte Growth Factor as a Target of MicroRNA-155 in Lung Fibroblasts: Implication in Epithelial-Mesenchymal Interactions. PLoS ONE.

[73] Qin S., Li B., Chen M., Qin M., Liu J., Lv Q. (2022). MiR-32-5p promoted epithelial-to-mesenchymal transition of oral squamous cell carcinoma cells via regulating the KLF2/CXCR4 pathway. Kaohsiung J. Med. Sci..

[74] Yu F., Zheng Y., Hong W., Chen B., Dong P., Zheng J. (2015). MicroRNA-200a Suppresses Epithelial-to-Mesenchymal Transition in Rat Hepatic Stellate Cells via GLI Family Zinc Finger 2. Mol. Med. Rep..

[75] Jimenez Calvente C., Del Pilar H., Tameda M., Johnson C.D., Feldstein A.E. (2020). MicroRNA 223 3p Negatively Regulates the NLRP3 Inflammasome in Acute and Chronic Liver Injury. Mol. Ther..

[76] Liu Y., Zhang H., Wang H., Du J., Dong P., Liu M., Lin Y. (2021). Long Non-Coding RNA DUXAP8 Promotes the Cell Proliferation, Migration, and Invasion of Papillary Thyroid Carcinoma via MiR-223-3p Mediated Regulation of CXCR4. Bioengineered.

[77] Leone V., D’Angelo D., Rubio I., de Freitas P.M., Federico A., Colamaio M., Pallante P., Medeiros-Neto G., Fusco A. (2011). MiR-1 Is a Tumor Suppressor in Thyroid Carcinogenesis Targeting CCND2, CXCR4, and SDF-1α. J. Clin. Endocrinol. Metab..

[78] Yang R.Q., Teng H., Xu X.H., Liu S.Y., Wang Y.H., Guo F.J., Liu X.J. (2016). Microarray Analysis of MicroRNA Deregulation and Angiogenesis-Related Proteins in Endometriosis. Genet. Mol. Res..

