# Supplementary material for: Coinfection of Dermal Fibroblasts by Human Cytomegalovirus and Human Herpesvirus 6 Can Boost the Expression of Fibrosis-Associated MicroRNAs
Source: Microorganisms. 2023 Feb 6;11(2):412. doi: 10.3390/microorganisms11020412 (PMC9958881; doi:10.3390/microorganisms11020412)
Supplement: Supplementary file 1 [file microorganisms-11-00412-s001.zip › microorganisms-2181479-supplementary.pdf]

Table S1. miRNA expression in single- and dual-infected cells (*).

	Time post-infection														
	0 d.p.i.			1 d.p.i.			2 d.p.i.			4 d.p.i.			7 d.p.i.		
miRNA ID	HHV-6A	HCMV	HCMV/ HHV-6A	HHV-6A	HCMV	HCMV/ HHV-6A	HHV-6A	HCMV	HCMV/ HHV-6A	HHV-6A	HCMV	HCMV/ HHV-6A	HHV-6A	HCMV	HCMV/ HHV-6A
hsa-let-7d-5p	1.21	1.05	−2.03	1.08	−1.13	−1.11	1.29	−1.04	1.37	2.08	3.34	2.95	−1	1.85	1.8
hsa-miR-1-3p	3.17	1.09	−1.37	1.01	1.12	−1.22	−4.13	−4.58	3.06	−3.16	11.18	5.82	3.43	39.18	107.3
hsa-miR-101-3p	−1.07	1.01	−1.43	−1.1	−1.22	−1.2	1.2	−1.03	1.91	−1.78	1.75	1.98	−2.29	2.27	1.86
hsa-miR-107	1.34	1.53	−1.58	1.02	−1.48	−1.45	−1.04	−1.38	1.22	1.96	2.17	2.88	−1.03	1.43	2.21
hsa-miR-10a-5p	−1.29	1.52	−3.12	−1.51	−2.26	−1.65	−1.56	−3.93	−1.15	2	1.78	1.99	−1.44	−2.43	−1.23
hsa-miR-10b-5p	−1.08	0.09	−1.82	1.77	1.23	1.33	1.23	1.01	1.68	2.05	2.09	3.11	−1.08	1.38	1.53
hsa-miR-122-5p	−2	−2.01	−1.37	1.01	1.12	−1.22	2.54	2.29	4.58	21.94	44.81	187.5	3.43	31.23	82.16
hsa-miR-125b-5p	1.03	−1.5	−1.83	1.11	−1.49	−1.51	−1.06	−1.17	1.23	1.76	2.41	2.26	−1.3	1.43	−1.01
hsa-miR-126-3p	−1.85	−1.89	−2.73	−1.3	−3.05	−3.44	1.01	1.59	1.02	2.17	2.92	4.47	−1.53	−1.28	2.82
hsa-miR-129-5p	3.54	2.58	−1.37	10.3	1.12	−1.22	−13.75	−15.23	3.45	1.81	1.66	65.72	−8.32	2.03	17.79
hsa-miR-132-3p	1.26	1.01	1.11	−1.95	1.09	1.94	−1.19	1.39	1.82	1.65	3.2	2.95	1.73	4.77	4
hsa-miR-133a-3p	−2	5.987	3.78	4.34	1.12	−1.22	2.54	13.44	15.52	9.86	26.76	10.46	15.93	20.55	13.84
hsa-miR-141-3p	−3.45	−2.32	−2.69	7.54	27.59	15.51	−9.42	1.36	−24.82	8.83	1.66	5.06	11.77	18.28	19.7
hsa-miR-142-3p	26.83	25.26	5.57	12.12	1.12	22.97	2.87	13.88	13.16	9.31	3.57	7.23	−22.9	16.9	−12.83
hsa-miR-143-3p	1.02	1.05	−1.44	1.42	−1.41	−1.02	1.01	−1.08	1.48	1.94	4.39	2.06	1.01	2.86	−1.23
hsa-miR-145-5p	1.05	1	−1.19	1.18	−1.37	−1.29	1.11	−1.24	1.21	2.52	−4.15	2.02	1.32	1.01	−9.83
hsa-miR-146a-5p	−1.06	−1.5	−1.81	1.22	1.13	1.24	−1.19	−1.86	1.46	−1.95	9.15	3.51	−3.3	8.25	2.74
hsa-miR-146b-5p	1.26	1.56	−1.32	1.25	1.23	−1.69	−1.23	−1.23	1.61	3.03	8.59	7.04	−1.58	3.05	3.44
hsa-miR-148a-3p	1.03	1.06	−1.34	1.5	−1.17	1.25	1.04	−1.26	1.59	−1.08	−2.07	2.47	−1.72	−1.84	1.46
hsa-miR-150-5p	36.63	25.98	52.51	15.74	1.12	75.2	1.03	15.4	23.45	30.54	20.19	54.25	−1.62	−2.89	4.46
hsa-miR-155-5p	−1.08	−1	−1.3	1.11	1.64	1.21	1.41	1.36	2.75	3.6	3.19	63.32	2.71	1.57	4.07
hsa-miR-15b-5p	1.71	1.56	1.04	1.97	1.45	2.15	1.21	1.06	2.01	1.91	1.13	3.42	1	−1.18	1.94
hsa-miR-16-5p	1.14	1.34	−1.45	1.27	−1.1	1.02	1.09	1.25	1.87	1.27	1.78	3.04	−1.68	1.67	1.61
hsa-miR-17-5p	−1.05	1.62	1.03	1.38	1.14	1.11	1.11	1.54	1.75	1.13	2.08	2.47	−1.29	1.39	1.91
hsa-miR-18a-5p	−1.01	−1.06	−2.63	1.33	−1.17	1.38	1.13	2.21	1.79	2.95	2.17	−5.11	−1.77	1.22	−3.22
hsa-miR-192-5p	−1.65	1.05	1.26	−1.5	1.37	1.04	1.41	1.06	3.1	1.44	4.98	9.82	−1.38	3.91	11.12
hsa-miR-194-5p	1.22	−1.09	−2.89	−1.09	−1.92	1.26	−1.31	1.07	1.37	1.86	3.01	−4.14	1.79	3.06	−2.14
hsa-miR-195-5p	−1.19	−1.02	−1.71	1.78	−1.2	−1.06	1.38	−1.08	2.25	1.16	3.26	4.79	−2.45	2.82	1.09
hsa-miR-196a-5p	1.02	1.03	−1.42	1.27	−1.27	1.02	1.28	1.16	1.44	2.5	2.46	1.79	1.3	1.5	−1.83
hsa-miR-199a-5p	1.29	1.02	−1.26	1.5	−1.16	1.06	1.1	−1.49	1.1	1.34	1.3	2.17	−1.53	1.37	−1.69
hsa-miR-199b-5p	1.08	1.01	−1.38	1.1	−1.45	−1.36	1.23	−1.31	1.25	−1.29	−1.53	3.1	−2.27	−1.53	2.88
hsa-miR-19a-3p	1.05	1.52	−1.66	1.28	1.08	1.07	1.41	1.17	1.96	−1.11	2.23	3.47	−1.81	1.7	5.78
hsa-miR-19b-3p	1.34	1.34	−1.57	1.57	−1	1.03	1.1	1.01	1.76	−1.18	2.38	69.24	−2.01	1.48	42.93
hsa-miR-200a-3p	−2	−1.98	−1.37	1.01	1.12	−1.22	2.54	2.29	−3.52	1.81	20.72	−8.04	−1.12	22.06	−3.14
hsa-miR-200b-3p	−2	−1.65	−1.37	1.97	23.2	−1.22	−1.56	−10.45	4.48	−22.4	1.45	337.25	1.42	2.16	1321.79
hsa-miR-203a-3p	−2	2.98	−3.61	35.32	1.12	−4.52	−14.12	2.04	−18.04	1.81	1.66	−1.14	3.43	108.15	−2.4
hsa-miR-204-5p	2.34	1.05	1.42	1.49	−1.41	1.55	−2.35	−1.02	1.44	2.75	9.82	1.51	1.32	7.76	2.16
hsa-miR-208a-3p	−2	1.03	−1.37	1.01	1.12	−1.22	2.54	2.29	1.98	2.38	1.66	2.49	3.43	2.25	1.28
hsa-miR-20a-5p	1.13	1	−1.6	1.22	−1.03	−1.08	1.38	1.41	1.91	1.18	1.71	−8.51	−1.53	1.68	−5.84
hsa-miR-211-5p	−2	−1.65	−1.37	1.01	1.12	−1.22	2.54	2.29	1.98	−6.24	−6.79	2.79	−3.51	−5.37	−35.37
hsa-miR-215-5p	1.42	−2.99	−15.47	2.33	2.64	3.89	−1.47	−2.96	3.98	2.66	4.09	12.37	1.55	2.54	3.01
hsa-miR-21-5p	1.16	1.52	−1.3	1.3	−1.03	−1.04	1.47	1.15	2.07	1.14	4.72	1.51	−1.34	4.36	2.16
hsa-miR-216a-5p	1.75	3.23	6.06	4.17	−21.61	−2.55	−3.8	−4.21	−4.86	1.81	18.55	1.51	3.43	2.25	12.16

hsa-miR-217	−2	−1.45	−1.37	1.01	1.12	−1.22	−7.91	−8.77	−10.11	1.81	1.66	1.51	3.43	2.25	2.16
hsa-miR-223-3p	14.61	22.54	9.23	−45.3	1.4	−55.67	2.54	25.66	−3.91	1.81	1.66	−5.05	3.43	7.42	−5.11
hsa-miR-23a-3p	1.05	1.35	−1.58	1.1	−1.24	−1.44	1.12	1.01	1.28	1.16	−1.96	3.12	−1.32	−1.44	−1.09
hsa-miR-25-3p	1.09	1.31	−1.17	1.01	−1.45	−1.49	1.52	1.15	2.2	3	3.15	2.35	1.16	1.48	1.32
hsa-miR-26a-5p	1.08	1.22	−1.31	1.44	−1.03	−1.02	1.31	1.11	1.47	2.13	2.84	3	−1.19	1.74	2.01
hsa-miR-26b-5p	−1.26	1.55	−2.4	1.51	−1.37	−1.16	−1.05	−1.12	1.54	1.52	2.41	2.46	−1.85	1.2	1.35
hsa-miR-27a-3p	1	1	−1.83	1.28	−1.23	−1.22	1.26	1.27	1.23	1.13	−1.61	1.51	1.67	2.39	2.16
hsa-miR-27b-3p	1.3	1.05	−1.53	1.47	−1.33	−1.16	1.23	−1.01	1.04	−1.11	−1.15	2.52	−1.6	1.31	−1.19
hsa-miR-29a-3p	1.39	1.09	−1.53	1.05	−1.24	−1.24	−1.02	−1.04	1.54	−1.48	1.18	2.25	−2.59	−1.07	1.16
hsa-miR-29b-3p	1.21	1.21	−2.15	1.39	1.15	1.17	−1.18	−1.58	1.53	−1.57	1.3	2.08	−3.33	−1.13	1.04
hsa-miR-29c-3p	1.39	1.35	−1.43	1.17	−1.09	−1.11	1.08	−1.04	1.52	−1.38	1.4	3	−2.47	1.02	−1.07
hsa-miR-302b-3p	−2	−1.68	−1.37	1.01	1.12	−1.22	2.54	2.29	1.98	1.81	1.66	1.51	3.43	2.25	2.16
hsa-miR-30a-5p	1.17	1.25	−1.66	1.44	−1.07	1.35	1.05	−1.17	1.42	1.03	2.15	2.17	−2.55	1.16	2.47
hsa-miR-31-5p	−1	1.22	−1.68	1.12	−1.26	−1.35	1.16	1.17	1.92	2.38	1.17	1.18	1.18	−1.12	−1.06
hsa-miR-324-3p	1.26	1.32	1	−1.03	−1.26	1.03	1.34	1.18	1.3	3.82	2.41	2.16	1.57	1.77	−1.23
hsa-miR-324-5p	1.1	−1.01	−1.19	−1.07	−1.5	−1.33	1.09	−1.16	1.3	1.41	1.56	1.51	−1.14	−1.19	2.16
hsa-miR-325	−2	−1.87	−1.37	1.01	1.12	−1.22	2.54	2.29	1.98	1.81	1.66	2.25	3.43	2.25	−1.2
hsa-miR-32-5p	1.73	1.24	1.84	1.32	1.45	1.93	1.13	−1.09	2.03	−3.5	1.18	5.68	−3.09	2.31	57.13
hsa-miR-328-3p	1.02	1.03	1.78	−1.3	−1.45	1.44	1.8	1.86	2.35	3.32	4.08	51.58	2.04	3	173.92
hsa-miR-335-5p	−1.01	−2.11	−3.01	1.2	−1.68	1.19	1.19	1.62	1.45	−1	1.62	2.3	−1.93	1.67	1.59
hsa-miR-338-5p	−2	−1.05	−1.37	−9.15	−8.26	−11.24	2.54	2.29	1.98	1.81	1.66	2.84	3.43	2.25	2.59
hsa-miR-34a-5p	1.23	1.16	−1.17	1.14	−1.1	−1.04	1.19	1.03	1.56	−1.1	1.4	2.7	−2.13	1.38	1.16
hsa-miR-372-3p	−2	−1.15	−1.37	1.01	1.12	−1.22	2.54	2.29	1.98	1.81	1.66	1.98	3.43	2.25	27.29
hsa-miR-375	−2	−1.12	−1.37	1.01	1.12	−1.22	2.54	2.29	59.17	1.81	1.66	23.71	3.43	2.25	114.48
hsa-miR-377-3p	1.04	1.05	−1.53	1.01	−1.42	1.03	−1.47	−2.1	1.2	−1.93	1.21	−9.04	−2.06	−1.77	28.7
hsa-miR-378a-3p	−1.3	1.32	−1.39	1	1.3	1.18	1.5	1.84	2.03	10.32	2.07	4.33	1.16	−1.31	1.38
hsa-miR-382-5p	1.4	1.21	−1.62	1.03	−1.26	1.03	−1.01	1.24	−1.09	2.97	1.06	4.73	1.22	−1.06	16.35
hsa-miR-449a	34.63	−2.98	−1.37	1.01	1.12	−1.22	2.38	−13.33	−35.23	−17.23	−18.74	−3.04	−10.75	−16.42	1.83
hsa-miR-449b-5p	7.32	−2.54	−1.37	1.01	1.12	−1.22	2.54	2.29	1.98	1.81	1.66	3.51	3.43	2.25	12.16
hsa-miR-451a	−2	−1.01	−1.37	−1.53	−1.38	−1.18	−1.95	−2.16	−2.49	6.08	13.13	1.89	1.86	193.02	2.47
hsa-miR-491-5p	−1.08	1.22	1.02	1.04	1.04	−1	1.57	1.27	4.6	1.89	1.68	10.5	−1.66	1.74	13.35
hsa-miR-5011-5p	−4.62	−4.99	−9.89	−92.51	−7.77	−33.05	2.95	−1.53	−8.26	6.57	1.17	1.52	1.26	6.71	2.19
hsa-miR-503-5p	1.03	1.02	−1.34	1.3	−1.02	−1.46	−1.02	−1.28	1.76	1.53	1.01	3.83	1.72	1.54	1.89
hsa-miR-5692a	−2	−1.23	−1.37	1.01	1.12	−1.22	2.54	2.29	1.98	1.81	1.66	6.59	3.43	2.25	11.1
hsa-miR-590-5p	1.73	1.54	−1.8	1.43	−1.09	−1.59	1.28	1.76	2.93	−1.37	1.48	2.9	−1.56	1.66	2.72
hsa-miR-661	−2	1.58	1.23	−1.29	1.55	4.21	−19.01	−1.83	−24.29	2.07	−2.61	12.48	1.17	9.7	23.61
hsa-miR-663a	−1.21	1.56	1.06	−1.41	−1.05	−1.33	1.86	2.47	−1.82	1.61	3.7	1.24	2.8	3.32	−1.35
hsa-miR-744-5p	−2.27	−2.65	−2.04	−1.23	−1.38	−1.3	−1.58	−1.53	1.2	2.85	−1.58	1.49	1.21	−1.93	2.18
hsa-miR-7-5p	1.36	−3.68	−7.31	1.64	1.57	1.16	1.82	2.09	2.71	2.49	5.75	3.74	1.6	1.3	11.87
hsa-miR-874-3p	−1.84	1.54	−1.8	−1.06	−1.19	−1.54	1.42	3.41	5.24	2.24	3.6	6.45	1.55	4.12	6.52
hsa-miR-92a-3p	−1.02	1.45	1.06	1.11	−1.05	−1.05	1.33	1.63	1.82	2.48	2.13	2.84	1.94	2.19	2.7
SNORD44 (hsa)	1.38	1.12	1.1	1.12	−1.04	−1.03	1.28	1.58	1.46	1.41	1.54	2.42	1.11	1.51	3.58
SNORD38B (hsa)	1.51	1.02	1.77	1.15	1.05	1.22	1.52	1.45	1.76	1.84	2.55	1.5	1.18	1.74	1.71
SNORD49A (hsa)	1.26	1.32	−1.03	1.26	1.03	1.17	1.47	1.38	1.69	2.1	1.81	1.85	1.44	1.23	1.24
U6 snRNA (v2)	1.52	1.45	1	−1.63	−1.29	1.08	1.09	1.21	1.18	−1.58	−1.73	1.52	−1.84	−1.35	1.69

(*) *Light red*, miRNAs up-regulated between 2 and 9.99 fold. *Red*, miRNAs up-regulated >10 fold. *Light blue*, miRNAs down-regulated between -2 and -9.99 fold. *Blue*, miRNAs down-regulated >-10 fold.
